# Case Report: Breaking the cycle: preventing diagnostic escalation in pediatric chronic functional abdominal pain

**DOI:** 10.3389/fped.2026.1844256

**Published:** 2026-06-18

**Authors:** Khayreddine Bouabida, Margarieta Okang

**Affiliations:** 1 UConn Health, University of Connecticut, Farmington, CT, United States; 2 Department of Pediatrics, University of Montreal Hospital Centre (CHUM), Montréal, QC, Canada

**Keywords:** abdominal pain, chronic functional abdominal pain, diagnostic escalation, disorder of gut-brain interaction, iatrogenic harm, median arcuate ligament syndrome

## Abstract

Chronic abdominal pain in children often stems from disorders of gut–brain interaction (DGBI), yet families may struggle to accept these diagnoses when tests are normal. This case describes an eleven-year-old boy with persistent epigastric pain after abdominal injury whose presentation was most consistent with functional abdominal pain–not otherwise specified (FAP-NOS) under the Rome IV criteria. Despite extensive negative evaluations, his family believed vascular pathology was responsible, leading to escalating interventions including celiac plexus block and median arcuate ligament release. Symptoms returned post-operatively, consistent with gut–brain interaction rather than structural disease. This case underscores the critical need for early, clear explanations of gut–brain mechanisms that validate symptoms and address caregiver concerns, preventing diagnostic escalation and iatrogenic harm while supporting functional recovery.

## Introduction

Chronic abdominal pain in children without identifiable structural disease is increasingly understood through the framework of disorders of gut–brain interaction (DGBI). These conditions are biologically grounded and reflect measurable disruptions in visceral sensitivity, gastrointestinal motility, central sensitization, autonomic regulation, and psychosocial modulation (1, 2). They explain how children can experience severe, persistent pain despite normal laboratory, radiologic, and endoscopic evaluation. DGBIs are recognized as positive, mechanism-based diagnoses rather than diagnoses of exclusion and represent a substantial global burden in pediatrics (1–6).

Among the abdominal pain–related DGBIs defined by the Rome IV criteria — which include irritable bowel syndrome (IBS), functional dyspepsia (FD), abdominal migraine (AM), and functional abdominal pain–not otherwise specified (FAP-NOS) — chronic functional abdominal pain is one of the most common presentations in pediatric gastroenterology (1, 2, 7). Children with these conditions frequently experience reduced school attendance, avoidance of physical activity, social withdrawal, and diminished quality of life, with levels of impairment comparable to many chronic organic gastrointestinal diseases (1–6). Despite this, diagnostic uncertainty remains common. Families often struggle to reconcile visible suffering with repeatedly normal test results. When evaluations consistently return normal, some caregivers interpret this not as reassurance but as evidence that something important has been missed, which can heighten anxiety and drive further investigation (3, 4). This mismatch between symptom severity and objective findings may contribute to diagnostic escalation and delay engagement with effective gut–brain–directed therapies (1–4).

The case presented here illustrates how these dynamics can unfold in practice. An eleven-year-old boy developed disabling epigastric pain after minor abdominal trauma. Over the subsequent year, he underwent extensive evaluation at multiple tertiary centers, none of which demonstrated structural disease. Persistent concern for an undetected vascular etiology nonetheless led to invasive interventions, including a celiac plexus block and surgical median arcuate ligament syndrome (MALS) release. His postoperative recurrence of symptoms despite technically successful surgery aligns with the variability, limited durability, and high rates of persistent pain described in pediatric evaluations for suspected MALS and vascular compression syndromes (6, 8–11). This clinical pattern is more consistent with a DGBI — specifically chronic functional abdominal pain — than with ongoing vascular pathology.

Drawing on this case and existing evidence, we examine how diagnostic anchoring, caregiver anxiety, and communication challenges contribute to escalation in pediatric chronic functional abdominal pain. We then propose a conceptual framework that models the non-linear progression of diagnostic escalation and identifies intervention points where clinicians can redirect care, prevent iatrogenic harm, and maintain a strong therapeutic alliance. Although this framework was developed in the context of chronic functional abdominal pain, the escalation dynamics it describes may be relevant to other abdominal pain–related DGBIs.

## Case presentation

“Alex” is a pseudonym used for readability and confidentiality. He is an eleven-year-old boy with a history of asthma who was admitted to our institution in November 2025 for evaluation of three days of non-bloody, non-bilious vomiting and one episode of loose stool. These symptoms occurred in the setting of a year-long history of persistent epigastric pain that began in late 2024 after a blunt abdominal injury sustained during play with an older sibling. Pain began shortly afterward and progressed to daily episodes of sharp epigastric discomfort rated between seven and nine out of ten. Episodes lasted from minutes to hours and were frequently triggered by walking, physical activity, and eating. Over time, he restricted oral intake because of the predictable association between meals and pain.

During the following year, Alex's parents sought multiple evaluations at tertiary centers, including two major academic hospitals. His cumulative diagnostic workup included abdominal ultrasound, hepatobiliary scintigraphy, upper endoscopy, contrast-enhanced computed tomography (CT) of the abdomen and pelvis, magnetic resonance angiography (MRA), an upper gastrointestinal (GI) series with small-bowel follow-through, and serial Doppler ultrasound examinations of the celiac and superior mesenteric arteries. All studies were normal or demonstrated only physiologic variation. These findings mirror the diagnostic uncertainty described in pediatric evaluations for suspected MALS, where objective correlates are inconsistent and structural abnormalities often do not explain symptoms (5, 6, 8, 9).

Despite reassuring evaluations, Alex's functional status declined. He withdrew from sports, avoided physical activity, missed school frequently, and became socially isolated. In pursuit of a structural explanation, further interventions were attempted. He underwent a celiac plexus block in late 2024 with only transient benefit. In January 2025, he underwent surgical median arcuate ligament release for presumed vascular compression. The pediatric literature describes substantial variability in postoperative outcomes following this procedure, with many children experiencing partial or temporary improvement despite technically adequate surgery (6, 8–10).

Alex initially noted improvement, but by March 2025 his pain returned to baseline. Follow-up MRA demonstrated a widely patent celiac artery without recurrent compression. This pattern of initial improvement followed by recurrence aligns with evidence that persistent symptoms after MALS surgery frequently reflect centrally mediated pain mechanisms rather than ongoing vascular pathology (10, 11).

On admission to our institution, Alex was well hydrated and hemodynamically stable. His physical examination showed localized epigastric tenderness without guarding, rebound, distention, or palpable masses. Laboratory studies, including hematologic and metabolic panels, were reassuring aside from a mildly elevated erythrocyte sedimentation rate (ESR) and minimally low phosphorus. Abdominal radiography demonstrated no obstruction and moderate stool burden.

His vomiting resolved within twenty-four hours of hydration, and he tolerated a regular diet. The gastroenterology team reviewed his full diagnostic history and concluded that his symptoms were most consistent with a disorder of gut–brain interaction, reflecting mechanisms such as visceral hypersensitivity and central sensitization (5, 6, 9). Specifically, his presentation met the Rome IV criteria for FAP-NOS, characterized by episodic or continuous abdominal pain that does not occur solely during physiologic events and is insufficient to meet criteria for IBS, FD, or AM, after appropriate evaluation has excluded other medical conditions (7). Pediatric surgery repeated dynamic Doppler ultrasonography, again showing no evidence of vascular compression, with concordant radiology interpretation.

Alex's parents remained concerned about an undetected vascular abnormality. His father expressed worry that symptom-directed therapies might mask clinically relevant information. The care team explained that symptom management would not interfere with further evaluation if warranted. Alex received one dose of dicyclomine — an anticholinergic agent selected for its antispasmodic properties targeting visceral smooth muscle — without adverse effects, but his parents perceived no meaningful change and declined ongoing treatment after discharge. A single dose is insufficient to assess therapeutic efficacy, and this was communicated to the family. Similar patterns of hesitancy toward symptom-directed therapy have been described in pediatric chronic pain when structural explanations remain central in the family's illness model (5, 6, 8).

Nursing documentation noted intermittent avoidance of food due to fear of triggering pain and moments when his father encouraged eating during clinical assessment. These observations were interpreted as reflecting the complexity of symptom expression in chronic pain rather than intentional behavior.

At discharge, Alex was clinically stable and tolerating oral intake. The team recommended outpatient psychological support, evidence-based DGBI therapies, possible neuromodulation, and coordinated follow-up in a multidisciplinary pain program. The family declined these recommendations and chose to continue evaluation elsewhere.

## Discussion

Alex's case highlights the clinical, relational, and ethical challenges that arise when a child presents with chronic functional abdominal pain — one of the most common abdominal pain–related disorders of gut–brain interaction (DGBI) — despite consistently normal diagnostic studies and caregivers remain anchored to a structural illness model. DGBIs are highly prevalent, biologically mediated, and often as disabling as organic gastrointestinal diseases, yet their lack of structural correlates makes them difficult for families to accept (1, 2). Mechanisms including visceral hypersensitivity, central sensitization, altered gastrointestinal motility, autonomic dysregulation, and psychosocial modulation can produce severe symptoms without detectable tissue injury (1–6).

### Symptom severity in the setting of normal evaluation

Alex experienced a progressive decline in daily functioning, school attendance, physical activity, and nutritional intake despite repeatedly normal laboratory, radiologic, and endoscopic evaluations. This pattern is characteristic of pediatric chronic functional abdominal pain, where symptoms reflect altered gut–brain processing rather than structural pathology (1–6). Normal test results may heighten caregiver concern when they appear inconsistent with the child's distress (3, 4). This underscores the importance of early education on gut–brain physiology and clear communication about the limited diagnostic value of repeating studies once red flags are excluded (2, 6).

### Trauma as an anchoring explanatory model

The temporal relationship between Alex's abdominal trauma and symptom onset provided a compelling but misleading explanatory anchor for his family. While sensitizing events such as trauma or infection can initiate altered gut–brain signaling, they do not necessarily indicate persistent structural disease (1). Families may conflate the triggering event with ongoing pathology, reinforcing diagnostic anchoring even when imaging repeatedly shows no abnormality.

### Diagnostic anchoring and procedural escalation

Anchoring toward structural explanations is common in pediatric chronic pain (4, 5). Structural diagnoses appear tangible and correctable, whereas DGBI may initially feel uncertain despite strong biological foundations (1–6). This dynamic contributed to Alex undergoing both a celiac plexus block and median arcuate ligament release. In children, MALS is uncommon, difficult to diagnose, and associated with variable outcomes (8–11). Systematic reviews show high risk of bias in most pediatric studies evaluating MALS treatment, and outcomes frequently do not correlate with vascular findings (6, 11).

Importantly, recent pediatric data demonstrate that comorbidities play a major role in postoperative outcomes. In a 2024 series of children undergoing robotic MALS release, 83 percent had comorbid depression or anxiety, 50 percent had postural orthostatic tachycardia syndrome (POTS), and 42 percent had mast cell activation syndrome (MCAS), and despite reported improvement in “MALS pain,” 64 percent continued to experience symptoms from other comorbidities (10). This reinforces that pursuing vascular explanations in children with complex pain presentations often fails to address the predominant centrally mediated mechanism.

Alex's temporary postoperative improvement followed by symptom recurrence aligns with evidence that persistent pain in suspected MALS most often reflects neural dysregulation rather than ongoing vascular compression (9–12). Consistent with this, the 2025 European Society for Paediatric Gastroenterology, Hepatology and Nutrition (ESPGHAN) and North American Society for Pediatric Gastroenterology, Hepatology and Nutrition (NASPGHAN) guidelines strongly recommend against surgery for abdominal pain–related DGBI and emphasize early, mechanism-based therapies rather than procedural escalation (13).

### Diagnostic escalation framework

**Figure 1:**
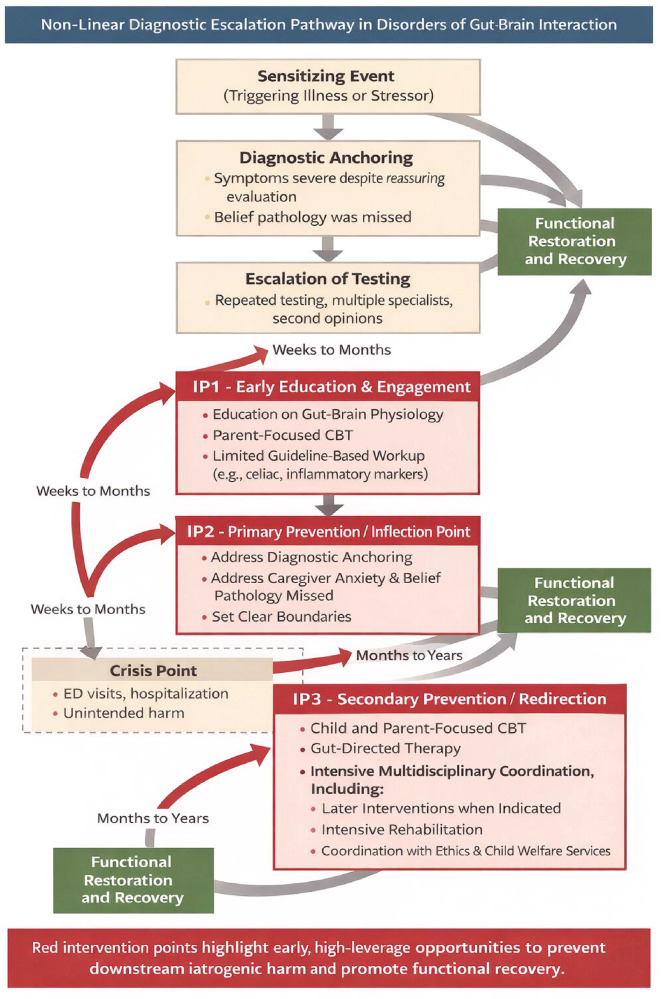
Preventing diagnostic escalation in pediatric DGBI: a conceptual framework.

The processes described above are synthesized in Figure 1, which illustrates a non-linear diagnostic escalation pathway from initial sensitizing events to crisis-level escalation, while identifying early intervention points where clinicians can redirect care and prevent iatrogenic harm.

To enhance clinical usability, we provide complementary components in Table 1 (clinical recognition) and Table 2 (intervention alignment).

**Table 1:** DGBI clinical and diagnostic recognition: stage-specific escalation patterns.

Escalation stage	Caregiver interpretation	System-level risk	Escalation signals
Initial Sensitizing Event	“Pain started after this event”	High focus on trigger	Symptoms persist beyond expected recovery
Symptom–Finding Mismatch	“Something important may have been missed”	Reduced reassurance; difficulty interpreting normal results.	Questions test validity; distress over normal results
Diagnostic Anchoring	“A structural diagnosis must exist”	Testing momentum; fragmented car	Multiple specialists; repeated imaging
Procedural Escalation	“A procedure seems necessary”	Iatrogenic harm; delayed functional therapy	Invasive procedures despite limited indication
Persistent Symptoms After Intervention	“It's been missed again”; confusion and frustration; renewed diagnostic search	Reinforced escalation cycle	Requests for more procedures or opinions
Crisis Point (Not Inevitable)	“Normal results don't explain the severity”; loss of trust; desperation	High care utilization and iatrogenic risk; psychosocial distress.	Hospitalizations; care fragmentation

Escalation stages are non-linear. Caregiver interpretations reflect common responses to diagnostic uncertainty. Signals help clinicians identify when to redirect care toward functional restoration.

DGBI, disorders of gut–brain interaction.

**Table 2:** DGBI intervention alignment: targeted strategies by intervention point.

IP	Target	Strategy	Actions
IP1 – Early Education	Misinterpretation of normal results	Education + parent-focused CBT	Positive diagnosis framing; guideline-based workup
IP2 – Inflection	Escalation-driving caregiver beliefs	Boundary setting + shared decision-making	Risk–benefit discussion; aligned team messaging
IP3 – Redirection	Entrenched escalation; functional decline	Multidisciplinary coordination	Unified care plan (pain, psych, GI, rehab); function-first goals
Later Interventions	System strain; care fragmentation	Ethical oversight and child well-being support	Case review; ethics consultation; safeguarding

Interventions vary according to escalation stage and may be applied flexibly based on clinical need.

IP, intervention point; CBT, cognitive behavioral therapy; GI, gastroenterology.

Although this framework was developed in the context of chronic functional abdominal pain, the escalation dynamics it describes — diagnostic anchoring, caregiver anxiety, and symptom–test mismatch — may be relevant to other abdominal pain–related DGBIs. However, prospective validation across DGBI subtypes is needed.

## Communication and diagnostic uncertainty

Communication is central in shaping family interpretation of incongruent symptoms and normal studies. Pediatric pain literature shows that clinician explanations can either reduce or amplify diagnostic uncertainty, and that validation of the child's suffering is essential for preserving trust (7, 14). Families may view normal test results as evidence that something has been missed unless clinicians provide clear, positive .explanations of DGBI mechanisms and describe why treatment should shift toward gut–brain–directed interventions (1–6, 13). Nursing observations of food avoidance and parental encouragement during assessments reflected the complexity of pain behaviors in DGBI and are consistent with documented patterns of heightened parental vigilance in pediatric chronic pain rather than intentional behavior (4, 15, 16).

## Evidence-based management and early intervention

Evidence supports a focused initial evaluation followed by early multimodal therapy for pediatric chronic functional abdominal pain (3, 11). Cognitive behavioral therapy (CBT) and gut-directed hypnotherapy have the strongest evidence base for improving pain, school attendance, and overall functioning (10, 12, 17, 18). Parent-focused interventions independently improve outcomes by reducing catastrophizing, protective behaviors, and unnecessary healthcare utilization (5, 16, 17). Early implementation of these strategies, combined with unified messaging and clear diagnostic boundaries, may prevent escalation and reduce iatrogenic risk.

## Ethical responsibilities and clinical boundaries

Clinicians have an ethical responsibility to prevent harm when diagnostic anchoring leads to pursuit of non-beneficial structural interventions (13, 19). Establishing clear limits on testing once comprehensive evaluation is complete is essential. Multidisciplinary review or ethics consultation may be appropriate when persistent diagnostic disagreement threatens patient well-being. These approaches support a consistent therapeutic message while protecting the child from unnecessary procedures.

## Framework interpretation

The proposed framework synthesizes interactions among neurobiological mechanisms, caregiver interpretations, communication patterns, and system factors that contribute to diagnostic escalation. It is hypothesis-generating rather than validated and reflects practice within a single tertiary center. Future studies should prospectively track families across the escalation stages to determine whether early intervention at identified points reduces procedural utilization, improves functional and psychosocial outcomes, and enhances family understanding and satisfaction with care.

## Synthesis

Alex's case demonstrates how symptom–test mismatch, caregiver anxiety, and communication gaps can drive diagnostic escalation in pediatric chronic functional abdominal pain and potentially other abdominal pain–related DGBIs. Reframing symptoms as manifestations of altered gut–brain physiology and initiating early, evidence-based therapy remain central to preventing chronicity and avoiding harm. Coordinated, mechanism-focused care and clear communication strategies are essential for interrupting escalation and restoring a trajectory centered on functional recovery.

## Strengths

This report proposes a novel framework that maps caregiver interpretations, diagnostic anchoring, and escalation patterns in pediatric disorders of gut–brain interaction. Grounded in established biopsychosocial pain models and Rome IV criteria (1–3, 13), the framework identifies critical inflection points where symptom–test mismatch and uncertainty may reinforce escalation. It is designed for multidisciplinary use and offers a practical tool to support early recognition, consistent communication, and prevention of unnecessary diagnostic and procedural interventions (5, 10, 12, 20–30).

## Limitations

This study is limited by its single-case, retrospective design and the absence of long-term follow-up, as the family elected to discontinue care at our institution and pursue evaluation elsewhere. This precludes assessment of whether the recommended DGBI-directed interventions would have altered the patient’s clinical trajectory. Despite this limitation, the case captures a complete escalation arc from initial presentation through surgical intervention and symptom recurrence, which is sufficient to illustrate the framework’s clinical relevance. The framework reflects practice within one tertiary academic center and may not generalize to all settings. Some observations may be subject to interpretation, introducing potential bias. Although grounded in current evidence on DGBI mechanisms and communication in pediatric pain (1–4, 12–14, 16, 19), the framework has not been prospectively validated and should be considered hypothesis-generating. Future research should test and refine this model across DGBI subtypes to determine whether early identification of escalation patterns improves clinical outcomes and reduces unnecessary interventions.

## Conclusion

Alex's case reflects the clinical and ethical challenges inherent in managing pediatric chronic functional abdominal pain, where severe symptoms persist despite reassuring diagnostic studies and caregivers remain anchored to structural explanations. His trajectory illustrates how symptom–test mismatch can intensify diagnostic uncertainty, reinforce anchoring, and drive escalation toward interventions with limited benefit and potential harm.

This case reinforces that DGBI is a positive, biologically grounded diagnosis requiring early, evidence-based treatment rather than repeated structural evaluation. Interventions targeting gut–brain pathways, delivered within a clear communication framework and supported by coordinated interdisciplinary care, offer the greatest opportunity to restore functioning and prevent chronicity.

The conceptual framework presented in this report maps common escalation patterns and identifies practical intervention points to help clinicians interrupt this trajectory. Ultimately, effective care depends not on identifying an elusive anatomic lesion but on restoring confidence, improving function, and guiding families toward a scientifically grounded and compassionate understanding of their child's condition.

## Data Availability

The original contributions presented in the study are included in the article/supplementary material, further inquiries can be directed to the corresponding author.
